# Transcriptome Sequencing (RNAseq) Enables Utilization of Formalin-Fixed, Paraffin-Embedded Biopsies with Clear Cell Renal Cell Carcinoma for Exploration of Disease Biology and Biomarker Development

**DOI:** 10.1371/journal.pone.0149743

**Published:** 2016-02-22

**Authors:** Oystein Eikrem, Christian Beisland, Karin Hjelle, Arnar Flatberg, Andreas Scherer, Lea Landolt, Trude Skogstrand, Sabine Leh, Vidar Beisvag, Hans-Peter Marti

**Affiliations:** 1 Department of Clinical Medicine, Nephrology, University of Bergen, Bergen, Norway; 2 Department of Clinical Medicine, Urology, University of Bergen, Bergen, Norway; 3 Department of Cancer Research and Molecular Medicine, Norwegian University of Science and Technology, Trondheim, Norway; 4 Spheromics, Kontiolahti, Finland; 5 Department of Pathology, Haukeland University Hospital, Bergen, Norway; 6 Department of Medicine, Nephrology, Haukeland University Hospital, Bergen, Norway; Institut National de la Santé et de la Recherche Médicale, FRANCE

## Abstract

Formalin-fixed, paraffin-embedded (FFPE) tissues are an underused resource for molecular analyses. This proof of concept study aimed to compare RNAseq results from FFPE biopsies with the corresponding RNAlater^®^ (Qiagen, Germany) stored samples from clear cell renal cell carcinoma (ccRCC) patients to investigate feasibility of RNAseq in archival tissue. From each of 16 patients undergoing partial or full nephrectomy, four core biopsies, such as two specimens with ccRCC and two specimens of adjacent normal tissue, were obtained with a 16g needle. One normal and one ccRCC tissue specimen per patient was stored either in FFPE or RNAlater^®^. RNA sequencing libraries were generated applying the new Illumina TruSeq^®^ Access library preparation protocol. Comparative analysis was done using voom/Limma R-package. The analysis of the FFPE and RNAlater^®^ datasets yielded similar numbers of detected genes, differentially expressed transcripts and affected pathways. The FFPE and RNAlater datasets shared 80% (n = 1106) differentially expressed genes. The average expression and the log2 fold changes of these transcripts correlated with R^2^ = 0.97, and R^2^ = 0.96, respectively. Among transcripts with the highest fold changes in both datasets were carbonic anhydrase 9 (*CA9)*, neuronal pentraxin-2 (*NPTX2)* and uromodulin (*UMOD)* that were confirmed by immunohistochemistry. IPA revealed the presence of gene signatures of cancer and nephrotoxicity, renal damage and immune response. To simulate the feasibility of clinical biomarker studies with FFPE samples, a classifier model was developed for the FFPE dataset: expression data for *CA9* alone had an accuracy, specificity and sensitivity of 94%, respectively, and achieved similar performance in the RNAlater dataset. Transforming growth factor-ß1 (*TGFB1)*-regulated genes, epithelial to mesenchymal transition (EMT) and *NOTCH* signaling cascade may support novel therapeutic strategies. In conclusion, in this proof of concept study, RNAseq data obtained from FFPE kidney biopsies are comparable to data obtained from fresh stored material, thereby expanding the utility of archival tissue specimens.

## Introduction

Clear cell renal cell carcinoma (ccRCC) makes up the majority of primary renal neoplasms with increasing incidence and considerable morbidity and mortality. Metastasis reflects a major cause of patient death [1, 2]. Renal cell cancer ranks among the ten most frequent cancers in women and men accounting for up to 2–3% of all adult cancers or malignancies [2–6].

The ccRCC is only curable by early surgical tumor removal. Thus, efforts to unravel molecular mechanisms of this disease for the search of prognostic markers and novel drug targets are important, e.g. by applying gene expression detection technologies to develop molecular signatures of disease progression.

In this study, we applied RNA sequencing (RNAseq), a method for measuring mRNA abundance based on next generation sequencing (NGS) technology. NGS can identify transcripts even at a low expression level and provides an increased dynamic range for gene expression measurements compared to microarrays [7, 8].

Current technologies for whole genome gene expression analyses are largely dependent on “high quality” RNA with low level of degradation. We wanted to test whether lower quality, partially degraded RNA obtained from archival formalin-fixed and paraffin-embedded (FFPE) renal tissues could serve as appropriate source of information.

The quality of RNA extracted from FFPE samples can vary widely among different specimens, or within different samples from the same specimen. RNA undergoes substantial chemical modification during formalin fixation, nucleic acids are cross-linked to proteins and RNA transcripts are degraded to smaller fragments [9]. Differences in formalin fixation methods and age of archival tissue samples add further variation to RNA quality. The Illumina TruSeq RNA Access Kit^®^ holds promise to overcome these challenges for RNA sequencing applications by isolating mRNA through a sequence-specific capture protocol resulting in reduced ribosomal RNA and enriched exonic RNA sequences. The TruSeq RNA Access library preparation kit was designed to ensure high quality RNA sequencing data from degraded FFPE samples and to allow comparison across samples that vary in quality.

Transcriptome sequencing of RNA from concurrently harvested FFPE and fresh stored kidney biopsies with subsequent analysis of transcripts and pathways underlying ccRCC in our patient group served as indication of the comparability of the two sources of RNA. The comparison to published data helped to estimate the biological and clinical plausibility of our results.

## Results

### Study design

This study includes 16 adult patients from Haukeland University Hospital with ccRCC undergoing partial (n = 10) or full (n = 6) nephrectomy between November 2013 and August 2014 (Table 1). Each patient donated four core biopsies, including two with ccRCC and two from adjacent non-affected tissue (“normal”). One pair of ccRCC and normal tissue per patient was then stored in FFPE, the other pair in RNAlater^®^. This paired design allows comparison of mRNA abundance level differences between ccRCC and normal in FFPE and in RNAlater^®^, and to evaluate the impact of storage condition on expression profiles using RNAseq.

**Table 1:** Characteristic patient features at the time of surgery. eGFR was calculated with the MDRD formula. The staging was performed based on the EAU Guidelines on renal cell carcinoma: 2014 update [43].

Patient number	Age, yr	Gender	BMI	Nephrectomy type	eGFR (ml/min/1.73m^2^)	TNM-stage	Size (mm)	Fuhrmann grade	Stage
9	70	Male	24	Partial	>60	pT1AcN0cM0	18	2	I
10	69	Male	34	Partial	>60	pT3AcN0cM0	15	2	III
11	37	Male	27	Partial	>60	pT1AcN0cM0	19	2	I
13	63	Male	24	Full	40	pT3AcN0cM0	69	4	III
15	68	Male	28	Partial	>60	pT1AcN0cM0	21	2	I
16	53	Male	33	Full	56	pT3bN0M1	100	2	IV
18	78	Male	27	Full	47	T3AcN0cM0	60	2	III
19	71	Female	22	Full	>60	pT2aN0cM0	90	1	II
21	53	Female	25	Full	55	pT1BcN0cM0	65	2	I
22	49	Male	25	Partial	>60	pT1BcN0cM0	50	2	I
24	69	Male	27	Partial	>60	pT1AcN0cM0	25	2	I
27	46	Male	31	Full	>60	pT2BcN0cM0	117	3	II
29	54	Female	29	Partial	>60	pT1AcN0cM0	15	2	I
31	67	Male	25	Partial	>60	pT1AcN0cM0	18	1	I
32	36	Male	23	Partial	>60	pT1AcN0cM0	18	3	I
33	48	Male	28	Partial	>60	pT1AcN0cM0	38	1	I

### Quality of Extracted RNA

To assess the quality of the 64 samples of extracted RNA we determined the Agilent RNA integrity number (RIN). Currently, the RIN is the most commonly used measure to determine RNA quality for gene expression analysis [10]. However, RIN values from FFPE samples are not a sensitive measure of RNA quality nor are they a reliable predictor of successful library preparation. Accordingly, previous investigators have used mean RNA fragment size as a determinant of RNA quality for the RNA sequencing library preparation (Illumina TruSeq RNA Access Kit^®^) when working with RNA obtained from FFPE tissues [11–13].

We have therefore also used the DV_200_ metric, the percentage of RNA fragments >200 nucleotides to evaluate the RNA quality according to the recommendation of the manufacturer and as described [11–13]. Using DV_200_ to accurately assess FFPE RNA quality, and by adjusting RNA input amounts, high-quality libraries can be prepared from poor-quality FFPE samples. In this respect, a sufficient DV_200_ value of as low as 30% was reported [13].

The mean Agilent RNA integrity number (RIN) and mean DV_200_ values (95% CI) were 5.7 (5.10–6.30) and 61% (58–64) for RNAlater^®^ samples and 2.53 (2.33–2.73) and 75% (72–79) for FFPE samples, respectively.

### Gene Expression (mRNA Abundance)

The number of detected genes, which passed an expression filter of more than 15 cpm in at least 8 samples per dataset, for FFPE was n = 9164 and for RNAlater^®^ n = 9205. Notably, about 94% of the genes in each dataset (n = 8893) were common to both FFPE and RNAlater^®^ datasets; correlation of the logarithmic fold change was R^2^ = 0.93, and correlation of the average expression R^2^ = 0.97, as shown in S1 Fig.

**Fig 1:**
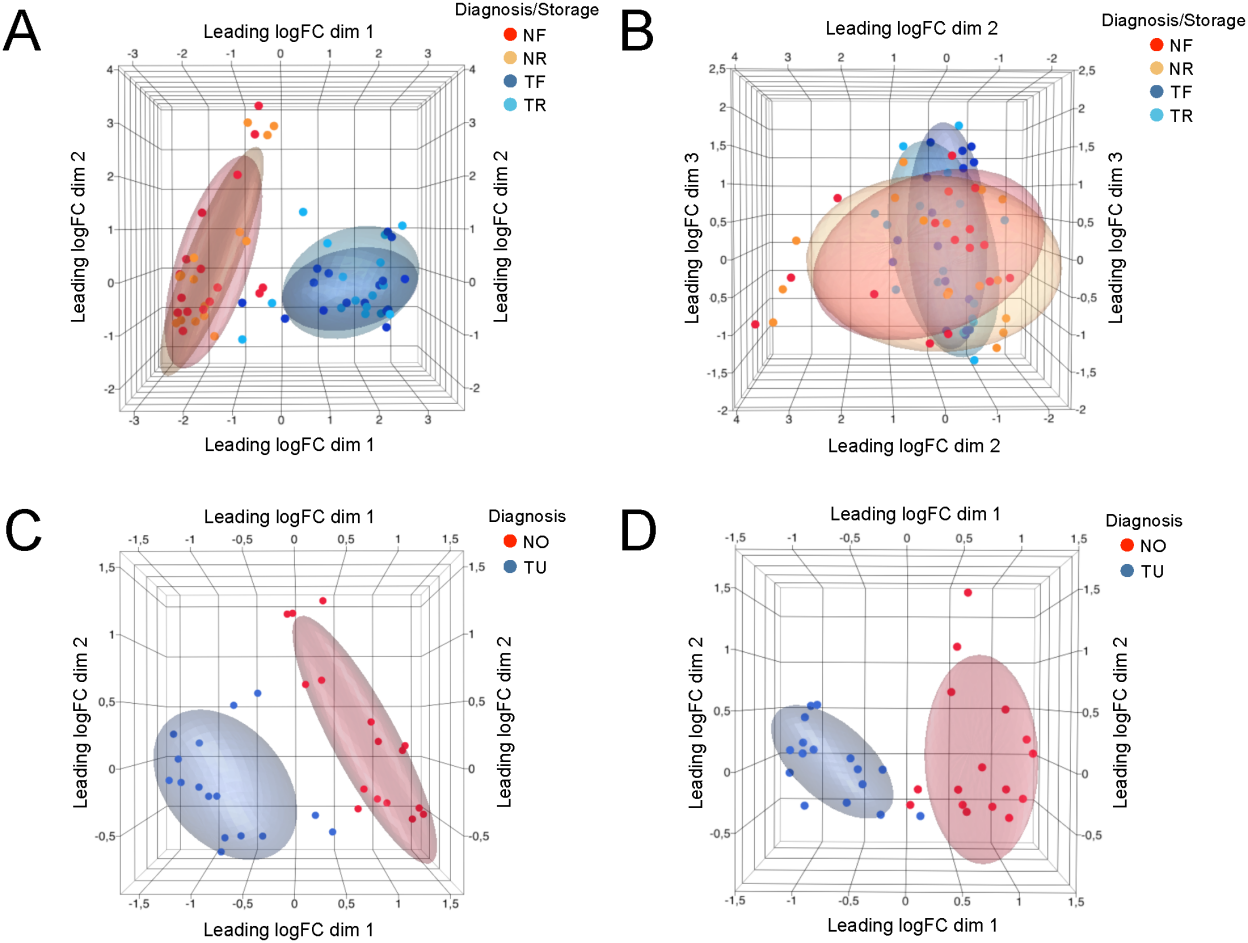
Multidimensional scaling (MDS) analysis of gene expression data. MDS analysis based on all commonly detected genes shows that samples segregate by diagnosis (A) and not by storage condition (B). Distances correspond to leading log-fold-changes between each pair of samples. MDS based on differentially expressed genes demonstrates less within-group variance compared to MDS with all detected genes in the RNAlater^®^ (C) and FFPE (D) datasets. *NF*: *Normal*, *FFPE; NR*: *Normal*, *RNAlater*^*®*^*; TF*: *Tumor*, *FFPE; TR*: *Tumor*, *RNAlater*^*®*^. *NO = Normal; TU = Tumor*.

To find sources of similarity in the dataset consisting of all 64 samples and the expression values of expression-filtered 8893 genes, we applied multidimensional scaling (MDS). Samples segregate into two large groups along the leading log-fold change in the dimension 1 of the MDS plot. The leading log-fold change is the average (root-mean-square) of the largest absolute log-fold change between each pair of samples. As deducible from sample annotation in Fig 1A, the major known factor explaining the similarity of biopsy samples was attributed to “Diagnosis” (i.e. tumor and normal). Storage condition (FFPE or RNAlater^®^) did not appear to cause sample segregation (Fig 1B).

In a next step, we identified for each dataset the genes with differential expression changes between ccRCC and normal, and compared the two sets. The FFPE dataset demonstrated 1367 differentially regulated genes and the RNAlater^®^ dataset 1418 genes (Benjamini-Hochberg adjusted p value ≤0.05, and abs FC ≥2); comparison of the non-tumorous, normal FFPE tissues versus the corresponding normal tissues from the RNAlater^®^ group revealed a very high concordance with only 37 differentially expressed genes (data not shown).

In the MDS analysis, plotting values for differentially expressed genes indicates less within-group variance compared to the analysis of all detected genes, and the shrinkage of log-fold changes indicates that some non-differentially expressed genes can have quite large fold changes (Fig 1C and 1D).

**Fig 2:**
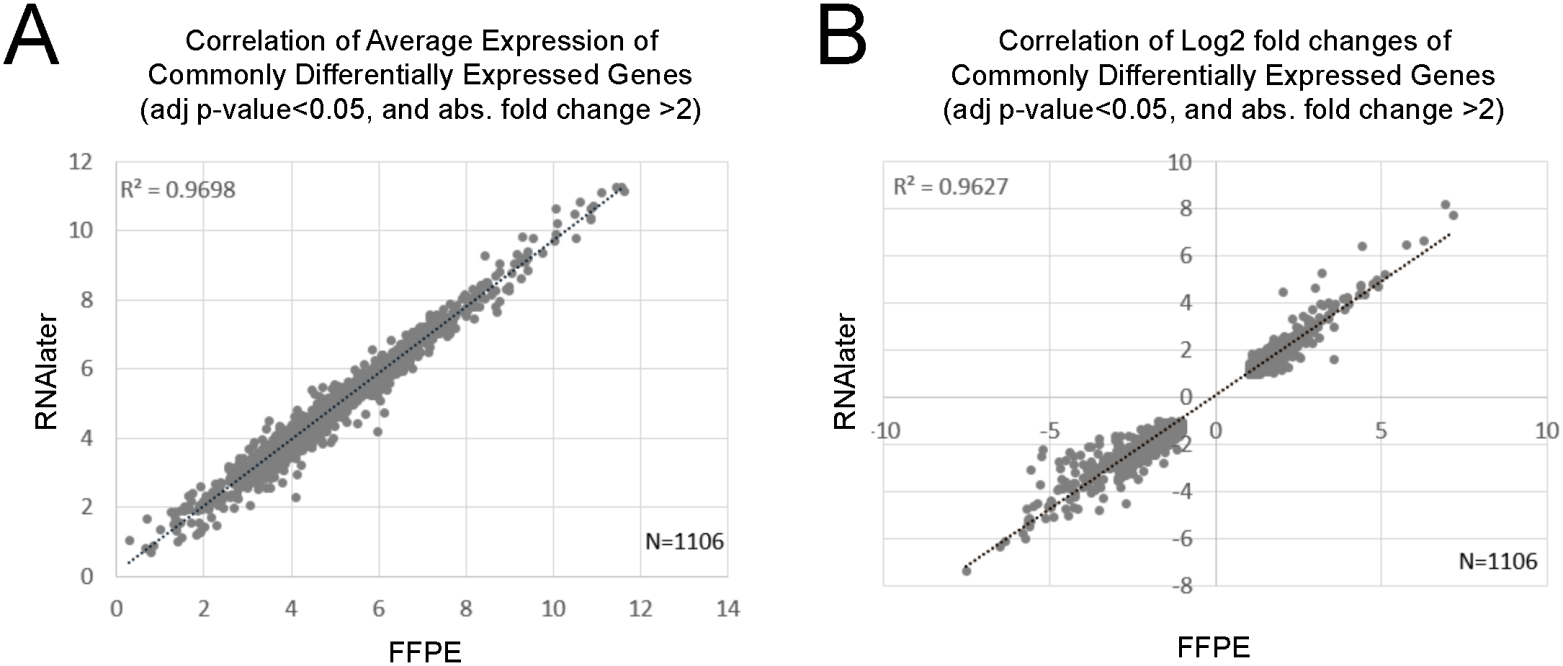
Correlation of gene expression data. The correlation of commonly differentially expressed genes is given with respect to (A) average expression and (B) log2 fold changes.

Each of these two datasets shared 1106 (about 80%) of differentially expressed genes with each other. The correlation of the average expression of these 1106 genes was R^2^ = 0.97 (Fig 2A). The log2 fold changes of these differentially expressed genes correlated by R^2^ = 0.96 (Fig 2B). All those genes in both datasets had the same direction of change. Table 2 shows the 20 most significantly affected genes with largest absolute fold changes in the FFPE dataset and the corresponding values of the RNAlater^®^ dataset; 17 of these 20 genes were differentially expressed in both datasets, 3 did not pass the expression filter in the RNAlater^®^ dataset. Amongst the 17 genes, 14 were among the top 20 ranking differentially expressed genes in the RNAlater^®^ dataset. Vice versa, all top 20 differentially expressed genes of the RNAlater^®^ dataset were differentially expressed in the FFPE dataset, 14 of which ranking among the top 20 in both datasets (not shown).

**Table 2:** Gene expression analyses. The 20 most up- or down-regulated genes in the FFPE data set with corresponding RNAlater^®^ values (upper panel), and the 20 most up- or down regulated genes in the RNAlater^®^ dataset with corresponding FFPE values (lower panel), filtered by adjusted p-value≤0.05. Rank indicates the rank of the gene within the list of differentially genes sorted by largest to smallest absolute fold change. 14 genes are shared between the two lists. *TU*: *tumour*, *NO*: *normal*, *FC*: *fold change*, *ND*: *not detected*, *did not pass the expression filter*.

**FFPE TU vs NO**							
		**FFPE**		**RNAlater**^**®**^		**rank**	
**Ensembl Gene ID**	**HGNC symbol**	**FC (TU vs NO)**	**adj. p-val.**	**FC (TU vs NO)**	**adj. p-val.**	**FFPE**	**RNAlater**^**®**^
ENSG00000169344	UMOD	-183,2	2,40E-07	-158,7	8,06E-08	1	3
ENSG00000106236	NPTX2	140,9	6,67E-07	220,1	2,29E-08	2	2
ENSG00000107159	CA9	121,2	5,50E-06	304,4	3,65E-09	3	1
ENSG00000074803	SLC12A1	-91,9	1,59E-07	-78,5	1,15E-07	4	7
ENSG00000169550	MUC15	-82,1	3,20E-07	-66,6	1,27E-06	5	8
ENSG00000142319	SLC6A3	76,6	2,17E-06	101,7	6,53E-07	6	4
ENSG00000169347	GP2	-57,2	1,13E-06	-52,7	4,52E-07	7	10
ENSG00000107165	TYRP1	-56,1	5,91E-06	ND	ND	8	ND
ENSG00000088836	SLC4A11	-54,2	1,14E-07	-62,7	2,16E-05	9	9
ENSG00000130822	PNCK	53,3	1,42E-06	92,0	1,89E-07	10	5
ENSG00000198691	ABCA4	-52,4	3,12E-07	ND	ND	11	30
ENSG00000165973	NELL1	-51,4	2,72E-07	-35,8	8,78E-07	12	16
ENSG00000186510	CLCNKA	-50,3	1,61E-08	-39,7	9,73E-08	13	13
ENSG00000215644	GCGR	-49,7	1,52E-07	-33,9	2,68E-06	14	18
ENSG00000164893	SLC7A13	-49,3	3,87E-04	-43,6	9,14E-06	15	11
ENSG00000138798	EGF	-47,9	1,43E-07	-37,3	2,26E-07	16	15
ENSG00000150201	FXYD4	-47,8	1,89E-05	-8,1	1,51E-02	17	134
ENSG00000184956	MUC6	-47,1	1,14E-05	ND	ND	18	ND
ENSG00000100362	PVALB	-45,7	5,83E-07	ND	ND	19	ND
ENSG00000130829	DUSP9	-45,0	7,90E-07	-24,4	1,56E-06	20	36
**RNAlater**^**®**^ **TU vs NO**						
		**RNAlater**^**®**^		**FFPE**		**rank**	
**Ensembl Gene ID**	**HGNC symbol**	**FC (TU vs NO)**	**adj. p-val.**	**FC (TU vs NO)**	**adj. p-val.**	**RNAlater**^**®**^	**FFPE**
ENSG00000107159	CA9	304,4	3,65E-09	121,2	5,50E-06	1	3
ENSG00000106236	NPTX2	220,1	2,29E-08	140,9	6,67E-07	2	2
ENSG00000169344	UMOD	-158,7	8,06E-08	-183,2	2,40E-07	3	1
ENSG00000142319	SLC6A3	101,7	6,53E-07	76,6	2,17E-06	4	6
ENSG00000130822	PNCK	92,0	1,89E-07	53,3	1,42E-06	5	10
ENSG00000185633	NDUFA4L2	87,6	6,30E-10	20,9	5,88E-06	6	50
ENSG00000074803	SLC12A1	-78,5	1,15E-07	-91,9	1,59E-07	7	4
ENSG00000169550	MUC15	-66,6	1,27E-06	-82,1	3,20E-07	8	5
ENSG00000088836	SLC4A11	-62,7	2,16E-05	-54,2	1,14E-07	9	9
ENSG00000169347	GP2	-52,7	4,52E-07	-57,2	1,13E-06	10	7
ENSG00000164893	SLC7A13	-43,6	9,14E-06	-49,3	3,87E-04	11	15
ENSG00000130208	APOC1	40,0	7,15E-09	9,1	6,01E-05	12	136
ENSG00000186510	CLCNKA	-39,7	9,73E-08	-50,3	1,61E-08	13	13
ENSG00000123610	TNFAIP6	37,8	2,98E-08	33,6	1,68E-07	14	26
ENSG00000138798	EGF	-37,3	2,26E-07	-47,9	1,43E-07	15	16
ENSG00000165973	NELL1	-35,8	8,78E-07	-51,4	2,72E-07	16	12
ENSG00000113889	KNG1	-34,9	7,04E-07	-35,6	5,31E-07	17	25
ENSG00000215644	GCGR	-33,9	2,68E-06	-49,7	1,52E-07	18	14
ENSG00000008196	TFAP2B	-32,5	4,04E-06	-29,9	7,56E-06	19	31
ENSG00000184661	CDCA2	32,4	1,77E-07	28,9	1,13E-06	20	33

### Immunohistochemistry

**Fig 3:**
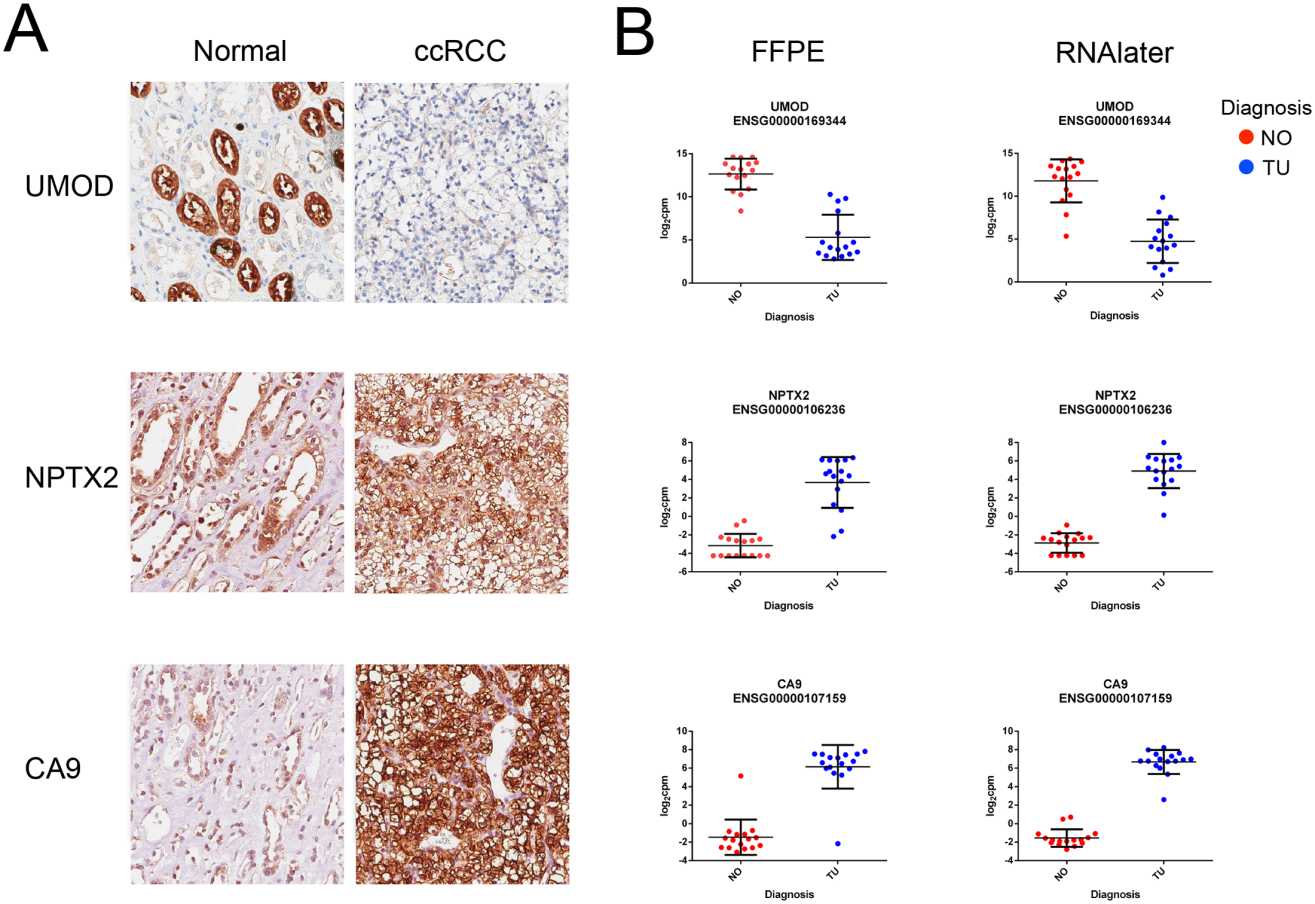
Immunohistochemistry and mRNA plots. (A) Immunohistochemistry of UMOD, NTPX2 and CA9. *Magnification x20*, *scale bar 50 μm*. (B) Respective mRNA abundance plots in the FFPE and in the RNAlater^®^ datasets.

Immunohistochemistry of the three most regulated genes according to Table 2 confirmed strong overrepresentation of neuronal pentraxin-2 (NPTX2) and carbonic anhydrase 9 (CA9) as well as the underrepresentation of uromodulin (UMOD) in ccRCC [14–16]. The results are depicted in Fig 3, which also presents respective mRNA abundance plots.

### Pathway Analyses

To test whether disease-relevant pathways have been captured in our experiment, we performed Ingenuity Pathway Analyses (IPA) of differentially expressed genes. 91 canonical pathways in the FFPE dataset and 109 pathways in the RNAlater^®^ dataset were affected (adjusted p-value≤0.05) with an overlap of 75%. The most affected pathways to a good extent reflect humoral and adaptive immune responses (Table 3). Sorting the pathways by smallest adjusted p-values, 12 of the top 20 in the FFPE dataset rank among the top 20 pathways in the RNAlater^®^ dataset.

**Table 3:** Pathway analysis. The 20 most affected canonical pathways in each NGS dataset with the corresponding values and ranks. Rank indicates the place of the pathway within the list of pathways sorted by largest to smallest –log(adjusted p-value). 12 of 20 pathways are shared between both datasets. *TU*: *tumour*, *NO*: *normal*, *FC*: *fold change*, *ND*: *not detected*, *did not pass the expression filter*.

**FFPE**	**-log(adj. p-value)**		**rank**	
	**FFPE**	**RNAlater**^**®**^	**FFPE**	**RNAlater**^**®**^
Antigen Presentation Pathway	13,90	9,13	1	3
Hepatic Fibrosis / Hepatic Stellate Cell Activation	13,90	14,60	2	2
LXR/RXR Activation	7,53	6,67	3	4
Leukocyte Extravasation Signaling	7,13	4,55	4	9
Coagulation System	6,78	6,59	5	5
Communication between Innate and Adaptive Immune Cells	6,60	3,58	6	17
Caveolar-mediated Endocytosis Signaling	6,54	3,69	7	12
Atherosclerosis Signaling	6,50	6,04	8	6
Dendritic Cell Maturation	6,50	4,18	9	10
Crosstalk between Dendritic Cells and Natural Killer Cells	6,31	3,62	10	14
Graft-versus-Host Disease Signaling	5,80	3,02	11	35
Complement System	5,78	4,55	12	8
Autoimmune Thyroid Disease Signaling	5,78	3,49	13	23
Virus Entry via Endocytic Pathways	5,78	2,92	14	38
OX40 Signaling Pathway	5,78	3,34	15	28
Intrinsic Prothrombin Activation Pathway	5,44	4,15	16	11
Allograft Rejection Signaling	5,44	3,49	17	25
Fcγ Receptor-mediated Phagocytosis in Macrophages and Monocytes	4,85	3,52	18	22
Granulocyte Adhesion and Diapedesis	4,36	2,74	19	47
iCOS-iCOSL Signaling in T Helper Cells	4,35	2,86	20	41
**RNAlater**^**®**^	**-log(adj. p-value)**		**rank**	
	**RNAlater**^**®**^	**FFPE**	**RNAlater**^**®**^	**FFPE**
EIF2 Signaling	14,60	ND	1	ND
Hepatic Fibrosis / Hepatic Stellate Cell Activation	14,60	13,90	2	2
Antigen Presentation Pathway	9,13	13,90	3	1
LXR/RXR Activation	6,67	7,53	4	3
Coagulation System	6,59	6,78	5	5
Atherosclerosis Signaling	6,04	6,50	6	8
LPS/IL-1 Mediated Inhibition of RXR Function	5,23	3,87	7	27
Complement System	4,55	5,78	8	12
Leukocyte Extravasation Signaling	4,55	7,13	9	4
Dendritic Cell Maturation	4,18	6,50	10	9
Intrinsic Prothrombin Activation Pathway	4,15	5,44	11	16
Caveolar-mediated Endocytosis Signaling	3,69	6,54	12	7
Ethanol Degradation II	3,62	1,49	13	77
Crosstalk between Dendritic Cells and Natural Killer Cells	3,62	6,31	14	10
Histamine Degradation	3,58	0,41	15	267
B Cell Development	3,58	3,15	16	32
Communication between Innate and Adaptive I Immune Cells	3,58	6,60	17	6
eNOS Signaling	3,58	2,78	18	41
Valine Degradation I	3,57	1,48	19	78
mTOR Signaling	3,57	ND	20	ND

### Comparison with Published Data

We compared our ccRCC gene expression changes with findings described in a recently published meta-analysis of ccRCC datasets [17]. All 10 most up-regulated genes and 7 of the 10 most down-regulated genes from Zaravinos et al. [17] were found in the present study and are differentially expressed in FFPE and RNAlater^®^ datasets (Table 4). The remaining genes did not pass our expression filter. The direction of fold changes was identical for all listed genes.

**Table 4:** Comparison of our gene expression data with data from literature [17]. Twenty genes with smallest p-values and largest absolute fold changes in a meta-analysis of five microarray studies are compared to the corresponding genes and their fold changes and p-values of the NGS datasets. The median fold changes and standard deviations for the meta-analysis are presented. All shown genes were differentially expressed in only 2 or 3 microarray datasets. Large standard deviations indicate a large spread of values in the individual microarray studies. 17 of the 20 genes were found differentially expressed in both NGS datasets, 13 of these with fold changes within the fold change range of the microarray meta-analysis. *ND*: *not detected*, *did not pass initial expression filter*.

Zaravinos et al. [17]			Eikrem et al. (present study)				
**Ten most significantly up-regulated genes**			**FFPE**		**RNAlater**^**®**^		
**HGNC symbol**	**Median fold change ± SD (TU vs NO)**	**p-value**	**Fold change (TU vs NO)**	**p-value**	**Fold change (TU vs NO)**	**p-value**	**Fold change within range of [17]**
NDUFA4L2	53,94±58,53	<0.01	20,9	4,09E-07	87,6	6.85E-14	yes
PLIN2	27,86±27,89	<0.01	4,6	2,82E-05	4,7	1,03E-04	yes
NNMT	20,86±9,84	<0.01	9,0	2,25E-07	15,8	5,47E-10	yes
ENO2	19,97±9,82	<0.01	6,3	7,10E-08	7,3	1,39E-10	no
AHNAK2	16,62±2,23	<0.01	12,2	8,66E-09	16,0	1,96E-08	yes
NETO2	15,8±13,8	<0.01	10,6	5,10E-10	11,7	5,06E-13	yes
CA9	14,48±4,40	<0.01	121,2	3,72E-07	304,4	3,17E-12	no
VWF	13,06±2,61	<0.01	4,9	3,84E-08	13,7	1,06E-09	yes
COL23A1	12,75±5,10	<0.01	22,1	6,99E-09	20,9	5,05E-09	no
EHD2	12,70±13,94	<0.01	3,9	2,26E-10	4,0	2,96E-08	yes
**Ten most significantly down-regulated genes**			**FFPE**		**RNAlater**^**®**^		
**HGNC symbol**	**Median fold change ± SD (TU vs NO)**	**p-value**	**Fold change (TU vs NO)**	**p-value**	**Fold change (TU vs NO)**	**p-value**	**Fold change within range of [17]**
ATP6V0A4	-19,70±32,54	<0.01	-10,4	5,39E-08	-7,4	2,28E-05	yes
CA10	-21,45±8,80	<0.01	ND		ND		
SLC12A3	-23,67±31,69	<0.01	-10,5	6,59E-05	-18,9	1,39E-06	yes
CLDN8	-27,11±95,38	<0.01	ND		ND		
SERPINA5	-35,45±32,90	<0.01	-13,7	3,34E-05	-16,4	9,39E-07	yes
KNG1	-38,45±51,67	<0.01	-35,6	1,15E-08	-34,9	9,64E-09	yes
KCNJ1	-50,79±59,09	<0.01	-2,4	1,48E-09	-2,1	1,48E-04	yes
RALYL	-53,58±11,02	<0.01	ND		ND		
CALB1	-103,68±156,0	<0.01	-12,00	1,45E-03	-8,4	7,88E-05	yes
NPHS2	-159,10±155,4	<0.01	-3,8	4,63E-03	-4,4	1,76E-03	no

**Fig 4:**
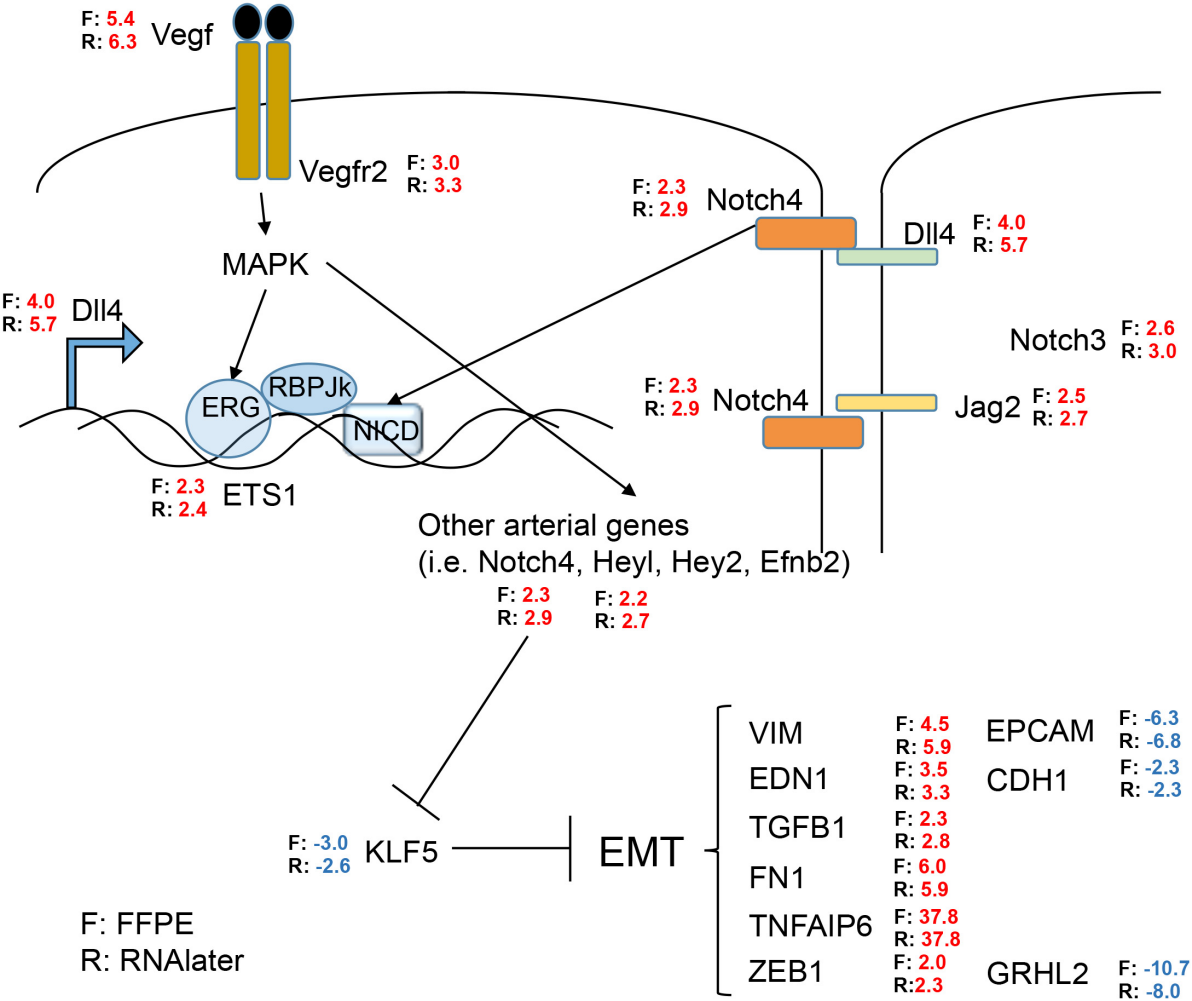
Pathway signature of VEGF and NOTCH mediated EMT in ccRCC. Comparison of gene expression data from the FFPE and from the RNAlater^®^ dataset with published results [20] and between themselves. *F = FFPE samples*, *R = RNAlater*^*®*^ *samples*, *Numbers = fold change of up-regulation (red) or down-regulation (blue)*.

We further compared the findings from the FFPE and the RNAlater^®^ datasets in relation to the known involvement of vascular endothelial growth factor (VEGF) in ccRCC [18, 19]. As demonstrated in Fig 4, many genes of the VEGF and NOTCH signaling cascades were retrieved in the FFPE and the RNAlater^®^ datasets with very similar fold changes and agreement in direction of changes. We can also confirm a link to epithelial to mesenchymal transition (EMT) by the overrepresentation of mesenchymal markers, e.g. vimentin (*VIM*), endothelin 1 (*EDN1*), fibronectin 1 (*FN1*), or transforming growth factor-β *(TGFB1)*, and underrepresentation of epithelial markers such as epithelial cell adhesion molecule (*EPCAM*) or E-cadherin (*CDH1*). The transcription factor grainyhead-like 2 (*GRHL2*), which inhibits EMT, is about 10 fold underrepresented [20].

**Fig 5:**
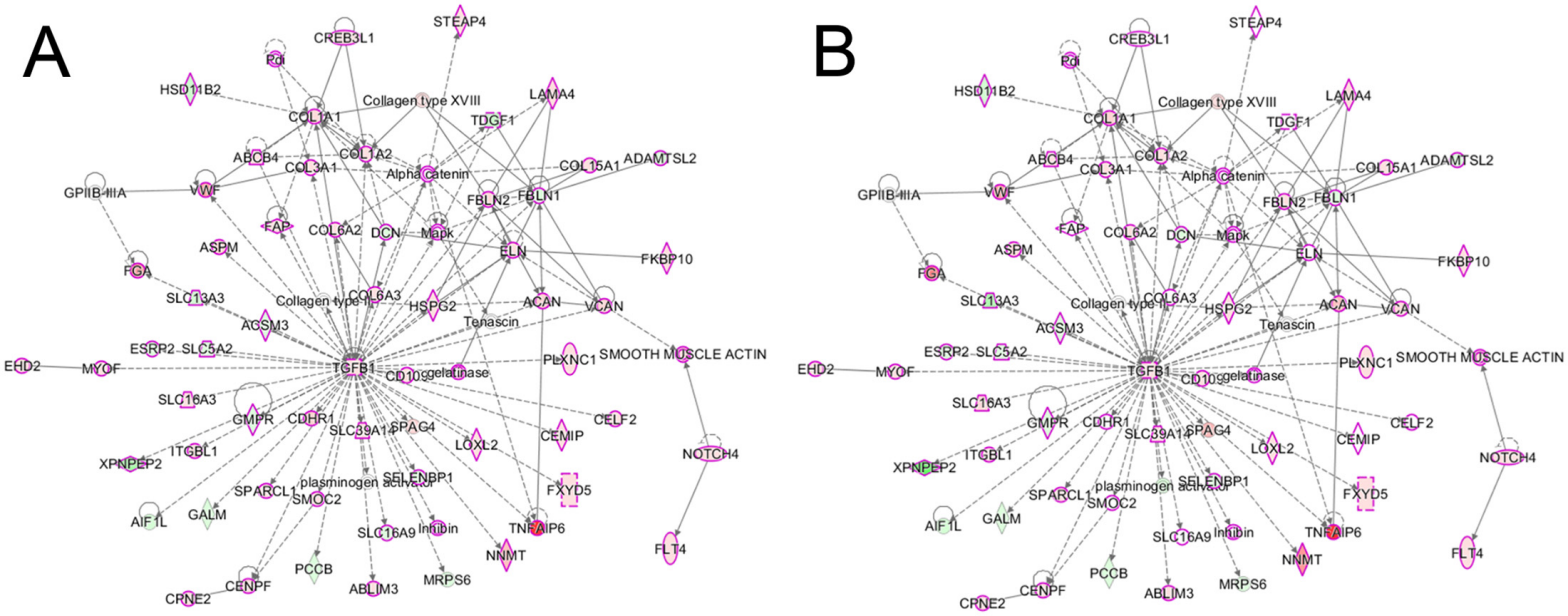
Gene network. The most differentially affected network with the central role of *TGFB1* in (A) FFPE samples and B) RNAlater data sets. *Proteins with cancer involvement are marked with purple outline*. *Red fill indicates overrepresentation of the gene in ccRCC*, *green indicates under-representation*. *Color intensity reflects range of fold change*.

IPA revealed *TGFB1* as one the most important regulator of gene expression in our ccRCC datasets, as shown in Fig 5. Of the 1367 differentially expressed genes in the FFPE dataset, the expression levels of 237 genes (17%) are influenced by *TGFB1* in the FFPE dataset (Fig 5A), and 253 of the 1418 (18%) differentially affected genes in the RNAlater dataset (Fig 5B). *TGFB1* itself was overrepresented 2.3 fold and 2.8 fold in the FFPE and the RNAlater dataset, respectively (Fig 4).

### Classifier Analysis

**Fig 6:**
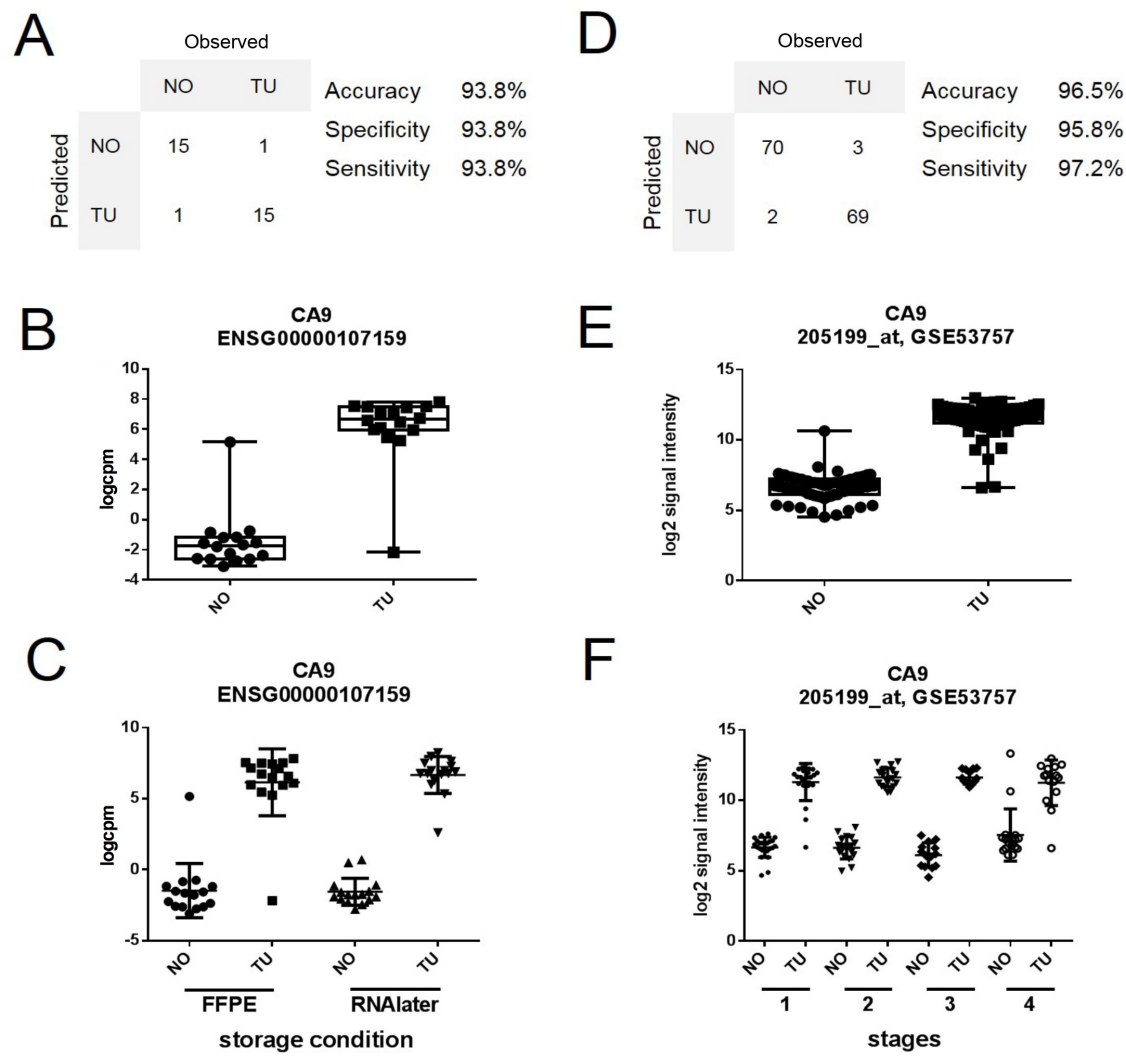
Development of a candidate marker for ccRCC. (A) Expression values of *CA9* correctly classified 30 of 32 samples in our FFPE dataset. (B) Whisker plot of expression value distribution in our FFPE dataset for *CA9*. (C) Scatterplot for the expression values of *CA9* in our FFPE and in our RNAlater dataset. (D) *CA9* expression values correctly classify 139 out of 144 samples in a microarray dataset of ccRCC (GSE53757). (E) Distribution of *CA9* expression values for normal (NO) and ccRCC tumor samples (TU) in the GSE53757 dataset. (F) Stratification of the expression values of overexpressed *CA9* into all four stages of ccRCC [14].

We further wanted to test whether the RNAseq data from the FFPE dataset could be used to develop a molecular classifier for ccRCC. Hence, in a proof of concept approach, we first selected 100 genes with the largest absolute fold change and smallest adjusted p-value among the group of differentially expressed genes in the FFPE dataset. To avoid overfitting, we initially tested the performance of classifier models with 15 or fewer genes, where we preferred those with few genes, as they would allow simpler testing in a clinical setting. *CA9* alone correctly classified 30 of 32 samples in the FFPE according to our annotation with an accuracy of 93.8% and area under the ROC curve (ROC AUC) of 0.96. Results of *CA9* from our patients are shown in Fig 6A–6C. One misclassified sample was a normal sample classified as tumor. However, importantly, this specimen contained some admixture of tumor tissue detected at a second look. The other misclassified sample from a different patient was a tumor sample with some adjacent tissue that was judged to be normal.

In the RNAlater^®^ dataset, the single gene classifier model assigned one sample with the histological classification “normal” to the group of tumor samples, yielding an accuracy ACC = 96.8%, AUC = 1.0, and a specificity of 93.8% and a sensitivity of 100%.

We then tested the single gene classifier model in an external dataset on a different technology platform. The publically available Gene Expression Omnibus (www.ncbi.nlm.gov/geo/) dataset GSE53757 contains Affymetrix HG-U133 microarray gene expression data from 72 human renal biopsies with four stages of ccRCC, and 72 matched normal samples [14]. The CA9-model correctly classified 139 of 144 samples independent of cancer stage (ACC = 96.5%, ROC AUC = 0.98). Results of this CA9 validation are shown in Fig 6D and 6E.

### Serum Analyses of CA9 Levels

Optimally, biomarkers such as the gene panel classifiers are further developed into clinically applicable tests. In our simulation study, we wanted to examine, whether CA9-assisted detection of ccRCC could be translated into a less-invasive clinical application going beyond the information obtainable from tissue samples. To that end, we measured CA9 protein in the serum of our patients with early T1a tumor stage and compared the results of these subjects with patient groups suffering from a more advanced disease, because a strong association between serum levels of CA9 with tumor stage has recently been reported [15].

Accordingly, ELISA analyses of serum samples from patients from our institution showed the following values: Increased CA9 levels (95% CI) of 237 (31–443) pg/ml in metastatic patients (n = 9), and of 112 (74–151) pg/ml in non-metastatic patients with high tumor load (tumors larger than 9 cm; n = 15), as compared to a concentration of 54 (26–83) pg/ml in subjects with T1a stage tumors (n = 14); p = 0.0069.

The between group analyses showed significant differences between patients with T1a tumor stage and either with high tumor load (p = 0.0031) or with metastases (p = 0.0158). The comparison between the latter two groups showed no significant difference.

Additional potential novel classifiers have been found, but await further examination and validation. For example, expression values of the highly up-regulated *TNFAIP6* (tumor necrosis factor, alpha-induced protein 6; Fig 4) showed similar performance as *CA9* in the FFPE, RNAlater^®^, and the microarray dataset (ACC = 96.9%, 96.7%, 94.4%, respectively). We are presently collecting more material and data to expand and confirm these findings.

## Discussion

Our proof of concept study compares transcriptome sequencing of RNA extracted from human renal biopsies of ccRCC and matched adjacent non-tumorous tissue; samples were preserved in two different storage conditions (FFPE and RNAlater^®^). High similarity of the two datasets indicates that archival FFPE-samples can be utilized in respective studies.

We chose RNAlater^®^ storage as the comparator. RNAlater^®^ is considered to be an excellent RNA stabiliser [21] and many studies show that RNA yields and gene RNA abundance with RNAlater^®^ are comparable to those obtained using frozen tissues [22]. Furthermore, the utilization of RNAlater^®^ is more practical allowing also decentralized tissue harvesting without special equipment [22, 23].

To the best of our knowledge, there has been no in depth report yet comparing matched RNAlater^®^ and FFPE storage conditions for parallel RNA sequencing and we are among the first to demonstrate the usability of the new Access kit (Illumina) also allowing low FFPE RNA amounts to generate RNA sequencing libraries. A related study has also demonstrated good concordance of RNA sequencing between the two storage conditions but has used different technology for only two renal cancers [24]. Obviously, the TruSeq Access kit is focused on studying mature mRNA levels in biological samples. A recent study has shown that other approaches, such as DSN (Duplex-specific nuclease)-seq and Ribo-zero-Seq can be used to investigate intergenic and intronic RNA species, reportedly giving information on slightly more mRNA species than polyA-enrichment methods, but at the expense of requiring more sequencing effort [25]. Where it is sufficient to study the human transcriptome coding regions, the TruSeq Access kit provides a cost-effective, highly reliable method, as our study shows.

Recent publications have studied the effect of storage time (up to 10 years) in FFPE on RNA quality and quantity, and the usability in mRNA expression experiments, both microarrays and RNAseq [26–28]. In concordance with our own unpublished data where we measured RNA quality and quantity from up to 30 year-old FFPE samples indicating their suitability for RNA sequencing, the publications agree that, RNA is still usable for RNAseq transcriptome studies although the RNA quality suffers with increasing time of FFPE-preservation.

Our approach is further supported by a recent publication showing that a newly developed exon capture RNAseq library preparation protocol for highly degraded RNA provided accurate estimates of RNA abundance, uniform transcript coverage and broad dynamic range investigating FFPE and flash frozen cancer tissues [29].

However, for the genome-wide detection of novel transcripts, whole exome enrichment of RNA might be a necessary additional step [30].

We detected a high degree of similarity between the gene expression results for the two datasets: 94% of the transcripts passing the initial expression filter were shared between the FFPE and RNAlater^®^ sample groups, 80% of differentially expressed genes were in common, and 75% of the differentially affected pathways were found in both datasets. The differences in gene expression can probably be mostly explained by the cell-composition variation of the respective biopsies. This well described intra-tumor heterogeneity precluded the detection of an even higher number of common, differentially regulated genes and pathways [31]. Also, the capture process during library preparation could be different depending on the RNA quality. However, the very high concordance between FFPE non-tumor, normal tissue *vs*. normal tissue stored in RNAlater^®^ further emphasizes the high similarity of the two data sets.

Despite some limitations, we have shown a striking similarity between the FFPE and the RNAlater^®^ datasets, maintaining biologically relevant information at large. Immunohistochemistry confirmed the three most regulated genes of both data sets. CA9 is essentially not expressed in the normal nephron but specifically in ccRCC [5]. Thus, CA9 is an extensively investigated biomarker of ccRCC and also a predictor of outcome following anti-VEGF therapy [19, 32]. In a microarray study with nine patients, UMOD was the gene with the strongest under-representation in RCC [16]. The over-representation of NPTX2 is in accordance with the literature [14].

We also show good concordance with microarray gene expression profiling studies of ccRCC (Table 4). Directions of gene expression changes between ccRCC and normal samples were identical for a set of differentially expressed genes in the microarray studies (14) and in the NGS studies. 17 of the 20 genes with largest absolute fold changes in the microarray meta-analysis were also differentially expressed in the NGS datasets (Table 4), and most fold changes were within the same range across the studies.

However, limitations and uncertainties in this comparison come from the large discrepancy in the fold changes detected in the microarray studies, and from the fact that all genes in the Table 4 were differentially expressed in only 2 or 3 of five microarray studies used in the meta-analysis. Different amplitudes in fold changes between the microarray dataset and the NGS dataset have been reported before [33]. The authors believe, one reason is that microarray probes might hit some, but not all, isoforms of a gene, and as a result the reported fold change of the probe set does not necessarily represent the expression change of the entire gene [33]. Furthermore, NGS is more sensitive in measurement of abundance differences of lowly or highly expressed genes. Microarrays reach a saturation level in the case of highly expressed genes, but NGS technology with its wider dynamic range of detection is more likely to detect fold changes. This may explain some of the fold change differences observed in the comparison of microarray and NGS data. Nevertheless, our dataset confirmed the trend of expression changes observed in microarray studies.

Our data also support and in part confirm novel therapeutic avenues, such as targeted at activated VEGF /NOTCH /DLL4 signaling cascades [18, 34–37]. The up-regulated NOTCH ligand Delta 4 (DLL4) is stimulated by VEGF and plays a role in tumor progression also predicting bad outcome [36, 38, 39]. EMT is augmented in our cancer data and is known to be a relevant feature in ccRCC [40]. Up-regulated *TGFB1* was the most significantly affected gene regulator in our study. Accordingly, *TGFB1* inhibition was shown to attenuate the invasive capacity of ccRCC cells [34]. However, potential cancer therapy targeted at *TGFB1* remains to be developed.

Classifier models consisting of features such as gene expression data in combination with a decision algorithm are powerful tools to support diagnostic and prognostic evaluation of patient data. Gene expression data for *CA9—*supplemented by CA9 serum protein data—showed an excellent performance both in our datasets and in an independent ccRCC microarray dataset. Thus, our data expand previous reports, which promote *CA9* as a diagnostic tool in ccRCC [5, 19, 41, 42].

Taken together, we show that in our hands RNAseq FFPE data are comparable to matched RNAlater^®^ data. We used the proof of concept data to explore and to confirm published biological findings, and findings which may be worth following up in larger cohorts, leading to possible novel therapeutic strategies, e.g. based on *TGFB1*-regulated genes, the *NOTCH* signaling cascade, and EMT. Also of note, FFPE tissues have the distinctive advantage that material designated for RNA sequencing can be concurrently investigated by light microscopy.

**Conclusions:** Our study opens the door to transcriptome analyses of the archival, FFPE stored tissues from patients with ccRCC and supports *CA9* as a potential marker for ccRCC.

## Materials and Methods

### Patients

Adult patients (n = 16) from Haukeland University Hospital with ccRCC undergoing partial (n = 10) or full (n = 6) nephrectomy and with the possibility to undergo biopsies for this project were included consecutively from November 2013 until August 2014. Patients had a mean age of 58.2±6.8 years (3 females and 13 males). Patients had pT tumor stages T1a (n = 10), T2a or b (n = 2) and T3a or b (n = 4) [43]; additional patient characteristics can be found in Table 1. The regional ethics committee of Western Norway has approved our studies (REC West no. 78/05). All participants provided written consent as requested by our ethics committee.

### Kidney Biopsies

Core biopsies have been obtained by O.E., L.L. and T.S. with a 16g needle from 16 patients undergoing (partial) nephrectomy in the operating room itself exactly at the time of surgery. Four paired biopsies from each patient with histologically-confirmed clear cell renal cell carcinoma (ccRCC) and adjacent non-tumorous (“normal”) tissue were either stored as FFPE tissue or in an RNA-stabilizing agent (RNAlater^®^, Qiagen, Germany). Total RNA was extracted with miRNeasy FFPE kit or miRNeasy micro kit (Qiagen), respectively.

### RNA Library Preparation and Sequencing

RNA sequencing libraries were prepared using TruSeq RNA Access library kit (Illumina, Inc., San Diego, CA, USA) according to the manufacturer`s protocol.

Initially total RNA concentration was measured using Qubit^®^ RNA HS Assay Kit on a Qubit^®^ 2.0 Fluorometer (Thermo Fisher Scientific Inc., Waltham, MA, USA). Integrity was assessed using Agilent RNA 6000 Nano Kit on a 2100 Bioanalyzer instrument (Agilent Technologies, Santa Clara, CA, USA) and the percentages of fragments larger than 200 nucleotides were calculated.

Thereafter, RNA samples (100 ng total RNA) were fragmented at 94°C for 8 minutes on a thermal cycler. First strand cDNA syntheses were performed at 25°C for 10 minutes, 42°C for 15 minutes and 70°C for 15 minutes, using random hexameres and SuperScript II Reverse Transcriptase (Thermo Fisher Scientific Inc., Waltham, MA, USA). In a second strand cDNA synthesis the RNA templates were removed and a second replacement strand was generated by incorporation dUTP (in place of dTTP, to keep strand information) to generate ds cDNA. AMPure XP beads (Beckman Coulter, Inc., Indianapolis, IN, USA) were used to clean up the blunt-ended cDNA from the second strand reaction mix. The 3`ends of the cDNA were then adenylated to facilitate adaptor ligation in the next step. After ligation of indexing adaptors, AMPure XP beads were used to clean up the libraries. In a first PCR amplification step, PCR (15 cycles of 98°C for 10 seconds, 60°C for 30 seconds and 72°C for 30 seconds) were used to selectively enrich those DNA fragments that have adapter molecules on both ends and to amplify the amount of DNA in the library. After validation of the libraries, using Agilent DNA 1000 kit on a 2100 Bioanalyzer instrument, the first hybridization step were performed using exome capture probes. Before hybridization a 4-plex pool of libraries were made, by combining 200 ng of each DNA library. The hybridization was performed by 18 cycles of 1 minute incubation, starting at 94°C, and then decreasing 2°C per cycle. Then streptavidin coated magnetic beads were used to capture probes hybridized to the target regions. The enriched libraries were then eluted from the beads and prepared for a second round of hybridization. This second hybridization (18 cycles of 1 minute incubation, starting at 94°C, and then decreasing 2°C per cycle) were required to ensure high specificity of the capture regions. A second capture with streptavidin coated beads were performed, followed by two heated wash procedures to remove non-specific binding form the beads. The enriched libraries where then eluted from the beads and cleaned up by AMPure XP beads prior to a second PCR amplification. The amplification step were performed by 10 cycles (98°C for 10 seconds, 60°C for 30 seconds and 72°C for 30 seconds) followed by a second PCR clean up using AMPure XP beads. Finally, the libraries were quantitated by qPCR using KAPA Library Quantification Kit—Illumina/ABI Prism^®^ (Kapa Biosystems, Inc., Wilmington, MA, USA) and validated using Agilent High Sensitivity DNA Kit on a Bioanalyzer. The size range of the DNA fragments were measured to be in the range of 200–650 bp and peaked around 270 bp.

Libraries were normalized to 22 pM and subjected to cluster and single read sequencing was performed for 50 cycles on a HiSeq2500 instrument (Illumina, Inc. San Diego, CA, USA), according to the manufacturer's instructions. Base calling were done on the HiSeq instrument by RTA 1.17.21.3. FASTQ files were generated using CASAVA 1.8.2 (Illumina, Inc. San Diego, CA, USA). Data are available in the repository Gene Expression Omnibus, http://www.ncbi.nlm.nih.gov/geo/query/acc.cgi?acc=GSE76207.

### Statistics and NGS Data Processing

We have a sample size of 64 samples, which is equivalent to 32 paired samples *(tumor samples vs*. *normal samples)*. Within both the FFPE and in the RNAlater dataset, we have 16 sample pairs *(tumors vs*. *normals)*. This sample size is sufficient to achieve a power of 0.85, where we apply a standard deviation of 0.7 of the expressed genes, an effect size of 2, and an alpha of 0.05 (R package RNASeqPower in https://www.bioconductor.org).

Assembly of reads and alignment of the contigs to the Human genome assembly GRCh38 was guided by Tophat and Bowtie. An empirical expression filter was applied, which left genes with more than 15 counts per million (cpm) in more than 8 samples per dataset. Comparative analysis was done using voom/Limma R-package. Differential gene expression was defined as Benjamini-Hochberg adjusted p-value ≤0.05, and an absolute fold change of ≥2. Pathway analysis was performed with Ingenuity Pathway Analysis (Qiagen, USA; version 24718999). The Ingenuity Knowledge Base information was used as reference set. Canonical pathways were sorted by smallest Benjamini-Hochberg-adjusted p-value.

Classifier analysis was performed with the KNNX Validation package in GenePattern (http://www.broadinstitute.org/cancer/software/genepattern). Leave-one-out method was used as internal cross validation method. Euclidean distance was used as distance measure, where three neighbors were considered. Data visualization was performed with JMP Pro 11 (www.sas.com), and Graphpad (www.graphpad.com).

### Histology and Immunohistochemistry

Immunohistochemistry was performed on 4 μm thick FFPE sections from the tumor and adjacent non-tumorous tissue. The following primary antibodies were used: Carbonic anhydrase IX (CA9, polyclonal, rabbit, NB100-417, Novus Biologicals), neuronal pentraxin 2 (NPTX2, polyclonal, rabbit, NBP1-50275, Novus Biologicals) and uromodulin (UMOD, polyclonal, rabbit, sc-20631, Santa Cruz Biotechnology). For positive controls, tissues with known positive reactivity were used, for negative controls the primary antibody was omitted. Slides were scanned with ScanScope^®^ XT (Aperio) at ×40 and viewed in ImageScope 12.

### ELISA for CA9 Serum Levels

CA9 serum concentrations of 38 patients was measured using the Quantikine Human Carbonic Anhydrase IX Immunoassay (R&D Systems, Minneapolis, USA, catalogue number DCA900) according to instructions of the manufacturer, but with an overnight incubation at 4°C after having added the serum. Results were assessed with the Kruskal-Wallis and Dunn’s test [44].

## Supporting Information

S1 Fig Correlation of the average expression of the commonly expressed genes in both FFPE and RNAlater datasets. Genes with an average expression of counts per million (cpm) >8 in at least 15 samples per dataset were considered.
(TIF)
