# Supplementary figures and images for: Transcriptome Sequencing (RNAseq) Enables Utilization of Formalin-Fixed, Paraffin-Embedded Biopsies with Clear Cell Renal Cell Carcinoma for Exploration of Disease Biology and Biomarker Development
Source: PLoS One. 2016 Feb 22;11(2):e0149743. doi: 10.1371/journal.pone.0149743 (PMC4764764; doi:10.1371/journal.pone.0149743)

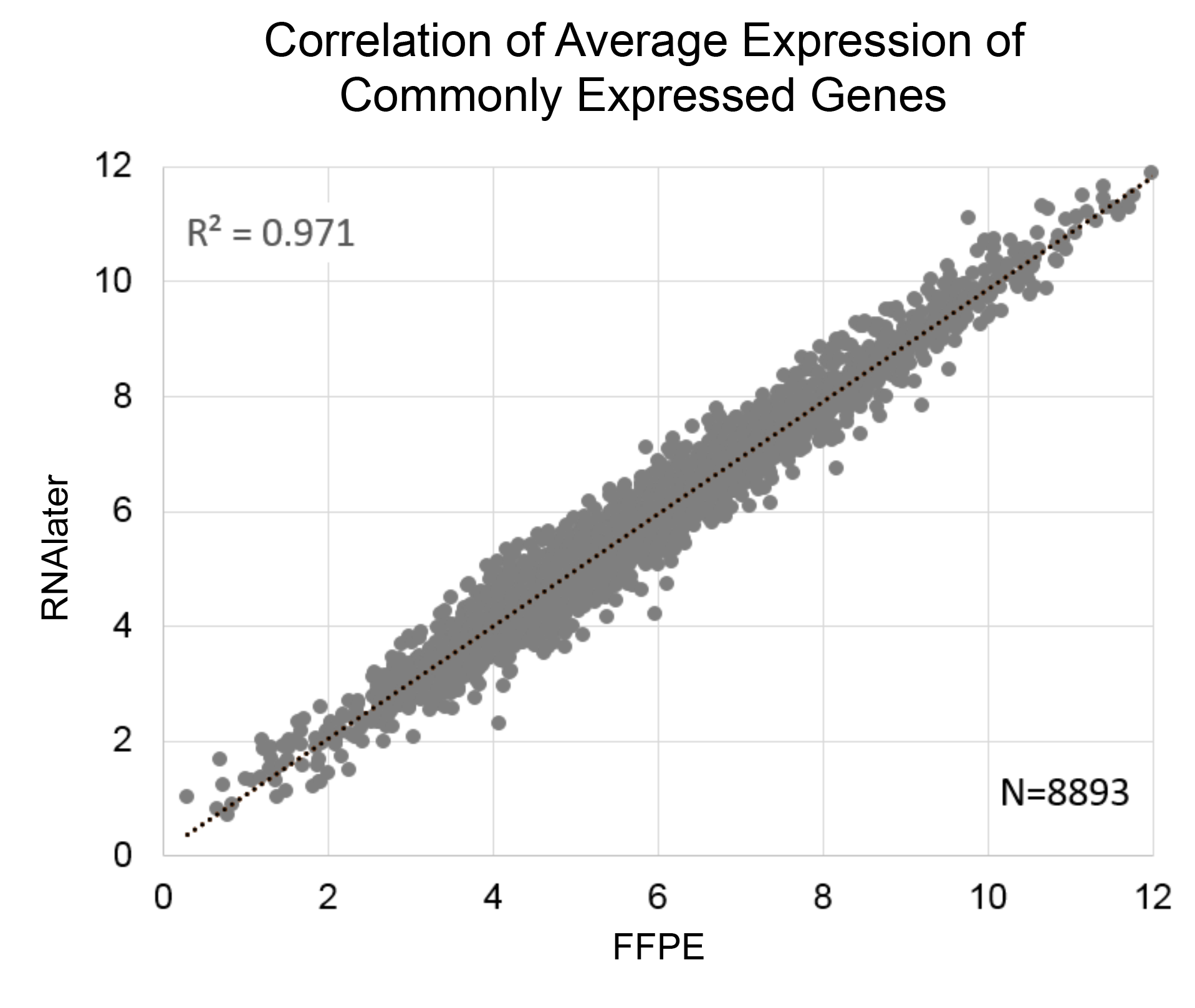

Supplement: S1 Fig — Genes with an average expression of counts per million (cpm) >8 in at least 15 samples per dataset were considered. (TIF) [file pone.0149743.s001.tif]
